# Abdominal Compartment Syndrome Following Massive Transfusion in Upper Gastrointestinal Bleeding: A Case Report

**DOI:** 10.7759/cureus.102969

**Published:** 2026-02-04

**Authors:** Hudson Martins de Brito, Tiago de Almeida Macruz, Gisele J Wakim, Livia Dahmen Rodrigues, Arman Dagal

**Affiliations:** 1 Department of Medicine, Federal University of Ceará, Fortaleza, BRA; 2 Department of Anesthesiology, University of Miami, Miami, USA

**Keywords:** abdominal compartment syndrome, blood component transfusion, blood transfusion, gastrointestinal hemorrhage, intra-abdominal hypertension

## Abstract

Abdominal compartment syndrome (ACS) is defined as sustained intra-abdominal pressure (IAP) exceeding 20 mmHg, leading to organ hypoperfusion and dysfunction. Although most cases arise from intra-abdominal or retroperitoneal hemorrhage, ACS may also occur after massive transfusion due to capillary leak and fluid overload. We describe an 82-year-old man presenting with upper gastrointestinal bleeding from a duodenal ulcer who required massive transfusion. During resuscitation, he developed progressive ventilatory failure with rising airway pressures and refractory hypoxemia. Intra-vesical IAP measured 85 mmHg, confirming severe ACS. Emergency decompressive laparotomy evacuated over two liters of serous fluid and revealed a markedly distended bowel filled with intraluminal blood but no hemoperitoneum. Ventilatory parameters improved immediately after decompression. Despite timely surgical intervention and intensive supportive care, the patient developed renal failure, acute respiratory distress syndrome (ARDS), methicillin-resistant *Staphylococcus aureus* pneumonia, and progressive neurological decline, ultimately leading to transition to comfort measures. Although such presentations are uncommonly reported, this case highlighted the critical importance of maintaining high clinical vigilance in patients undergoing massive transfusion, particularly when acute respiratory deterioration occurs.

## Introduction

Upper gastrointestinal bleeding (UGIB) is among the most common gastrointestinal diagnoses requiring hospitalization in the United States, accounting for more than 300,000 hospitalizations annually as the primary diagnosis [1], and management is determined by the underlying etiology. However, regardless of the cause, the initial approach prioritizes hemodynamic stabilization [2,3]. In patients with exsanguinating hemorrhage, massive transfusion protocols (MTP) represent a mainstay of resuscitation, although they are associated with several potential complications [4].

Intra-abdominal hypertension (IAH) is one such complication associated with polytransfusion. It is defined by a sustained or repeated pathological elevation in intra-abdominal pressure (IAP) ≥ 12 mmHg measured via the trans-bladder method, and graded in stages I-IV based on the values [5]. Physiopathologically, IAH can arise from an increase in intra-abdominal volume, such as ascites and intraluminal hemorrhage, and/or from a reduction in abdominal wall compliance, as in patients with sepsis and abdominal wall edema [6]. Treatment is individualized and targets the underlying, often multifactorial, factors that lead to increased IAP.

Complementary, abdominal compartment syndrome (ACS) is defined as sustained IAP > 20 mmHg, accompanied by recent onset organ dysfunction, which may result in severe impairment of the renal, gastrointestinal, cardiovascular, and respiratory systems [5].

Most cases of ACS result from intraperitoneal or retroperitoneal hemorrhage [7], while cases secondary to organ edema or ascites after massive resuscitation and reperfusion injury are less frequently described, particularly their progression in non-trauma patients. Diagnosis and treatment are typically performed in surgical or intensive care settings, but anesthesiologists are often the first to identify the ventilatory and hemodynamic effects during intraprocedural management.

This report highlights the case of an elderly patient with UGIB who developed refractory ventilatory failure due to critical ACS caused by both abdominal capillary leakage in the context of multiple transfusions and intestinal distension due to intraluminal hemorrhage. We emphasize the importance of anesthesiologists recognizing early ventilatory patterns suggestive of increased IAP during massive resuscitation. Written informed consent for the publication of this case report was obtained from the patient’s legal representative.

## Case presentation

We present an 82-year-old male with a body weight of approximately 68 kg, a height of 167 cm, and a body mass index (BMI) of 24.4 kg/m², with no known past medical history. He presented to the emergency department with abdominal pain, hematemesis, and a history of chronic melena and was found to have severe anemia (Hb 5.3 g/dL). Initially, the patient was hemodynamically stable, and procedures for UGIB were immediately initiated, with NPO, proton pump inhibitors (PPI), octreotide, ceftriaxone, isotonic crystalloid infusion, and transfusion of three units of packed red blood cells (PRBCs).

During the investigation, after 24 hours from the patient’s hospital admission, the patient underwent abdominal and pelvic computed tomography (CT scans), revealing no acute intra-abdominal pathology. An esophagogastroduodenoscopy (EGD) on the following day demonstrated Los Angeles grade A esophagitis and a duodenal ulcer in the second portion with a visible Forrest IIa vessel. The attempted bipolar cautery failed to achieve a thermal effect, and further intervention was withheld to avoid provoking hemorrhage. The procedure was concluded with no active bleeding and minimal blood loss. Over the remainder of the day, his course remained stable, and a transfer to the medical ward was being considered.

Approximately 10 hours after the EGD, the patient developed abrupt clinical deterioration, with altered mental status and complete disorientation to person, place, time, and situation, accompanied by hypotension. The nasogastric tube (NGT) returned about 350 mL of fresh blood, and initial arterial blood gas (ABG) demonstrated mixed acidosis, with a concomitant reduction in hematocrit, consistent with significant ongoing hemorrhage (Table 1 - ABG 1). The patient was subsequently intubated and transferred to the Medical Intensive Care Unit (MICU), where an MTP was initiated along with escalating vasopressor support, without additional large-volume crystalloid boluses. The transfusion strategy was initially guided by a 1:1:1 ratio of PRBC, fresh frozen plasma (FFP), and platelets, but was later adjusted according to blood product availability and thromboelastography (TEG) findings.

**Table 1:** ABG parameters recorded throughout the procedural interventions in the reported case ABG: arterial blood gas; BE: base excess; HCO₃⁻: bicarbonate; Hct: hematocrit; NR: not recorded; PaCO₂: partial pressure of carbon dioxide; pO₂: partial pressure of oxygen

Parameter	Reference value	ABG 1	ABG 2	ABG 3	ABG 4	ABG 5
pH	7.35-7.45	7.16	6.98	7.14	7.14	7.37
pO₂	80-100 mmHg	315	289	NR	NR	181
PaCO₂	35-45 mmHg	47	55	72	53	41
HCO₃⁻	22-26 mmol/L	17	12	24	26	24
BE	-4 to +2 mmol/L	-11	-19	NR	NR	-2
Hct	36-50%	26%	30%	NR	NR	27.8%

The surgery team determined that operative intervention carried prohibitive mortality without prior bleeding control, and the patient was sent to interventional radiology (IR) for angiography and embolization, about four hours after initial clinical deterioration. Between the initiation of the MTP and the patient’s transfer to the IR suite, a total of 17 units of PRBCs, 11 units of FFP, five units of platelets, and five units of cryoprecipitate had been administered.

**Figure 1:**
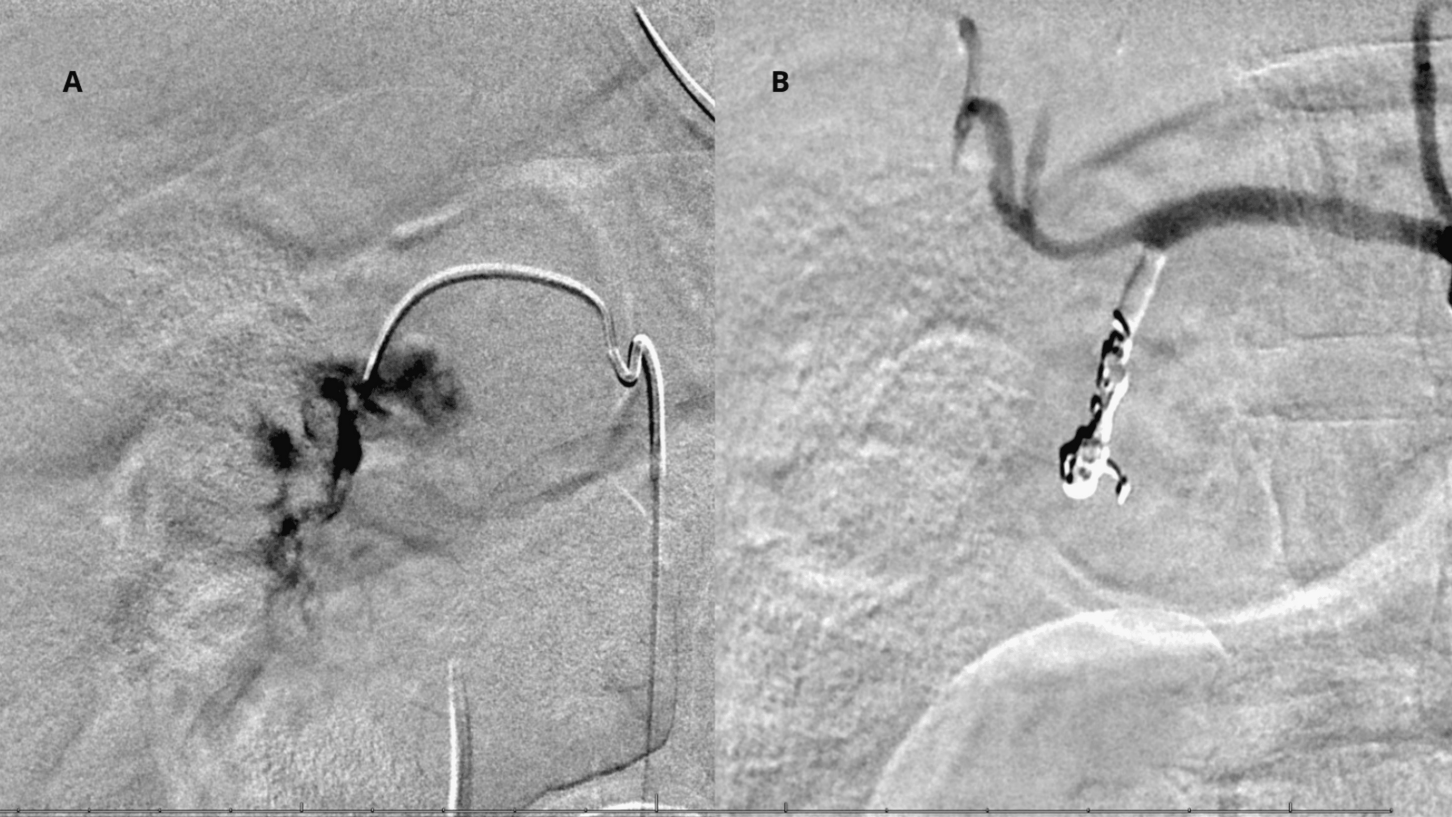
Gastroduodenal artery extravasation and embolization (A) Active extravasation of the gastroduodenal artery before embolization. (B) Gastroduodenal artery following successful embolization.

On arrival for angiography, the patient was mechanically ventilated in volume-controlled ventilation (VCV) mode with a tidal volume of approximately 400 mL (corresponding to 6.3 mL/kg of predicted body weight), a respiratory rate of 18 breaths per minute, and a positive end-expiratory pressure (PEEP) of 5 cmH₂O, at the expense of a peak pressure of 41 cmH₂O. A new ABG analysis (Table 1 - ABG 2) revealed severe mixed acidosis with worsening coagulopathy, and he received an additional four units of PRBC, two units of FFP, and two units of platelets. During angiography, active extravasation from the gastroduodenal artery (GDA) was confirmed, and a selective/subselective embolization was successfully performed (Figure 1).

However, progressive ventilatory failure ensued, with peak pressures rising to 60 cmH₂O despite reduced tidal volumes (200-300 mL). A new ABG (Table 1 - ABG 3) showed acidosis with severe hypercapnia. Desaturation followed (SpO₂ 80-85% on FiO₂ 100%) until complete cessation of mechanical ventilation was necessary due to extreme ventilatory pressures. Manual ventilation via bag-valve restored ventilation and oxygenation (SpO₂ 90%), suggesting abdominal compartment effect.

At that stage of the clinical course, the patient exhibited a sustained positive fluid balance from hospital day 1 through day 3, with a cumulative net positive balance of 4,562 mL, which included approximately 3,625 mL of crystalloid and 250 mL of human albumin administered for volume expansion in addition to blood products.

Given refractory ventilatory impairment, intra-vesical IAP measured 85 mmHg, diagnostic of severe ACS. An emergency midline decompressive laparotomy was performed in the IR suite, following approximately eight hours of MTP. Upon entry, more than 2 L of serous fluid were released, and the bowel appeared markedly distended, containing intraluminal blood. No hemoperitoneum or pneumoperitoneum was identified, and the abdomen was left open with two Jackson-Pratt (JP) drains. Ventilatory mechanics improved immediately (peak pressures ~40 cmH₂O, SpO₂ 100%), as demonstrated in Table 1 (ABG 4).

About six hours after laparotomy, postoperatively, the patient remained sedated and paralyzed under protective ventilation and low-dose vasopressors. After a few hours, a new ABG (Table 1 - ABG 5) showed marked improvement. At re-exploration, no active intra-abdominal bleeding was seen. A temporary closure was re-established, and another takeback was scheduled based on clinical course and ventilatory tolerance.

On the following day, approximately 30 hours after the initial laparotomy, the abdomen was re-explored, irrigated, and definitively closed at the fascial level. Peak pressures remained stable post-closure.

Immediately after the procedure, the patient was transferred to the MICU, and during his initial stay, the pressors were discontinued, but the patient progressed with high-output NGT and JP drainage and declining renal function. After two days, he was restarted on high-dose pressors, and renal function worsened. A chest CT showed bilateral ground-glass opacities with effusions, and bronchoalveolar lavage grew methicillin-resistant *Staphylococcus aureus* (MRSA). At that time, the neurologic exam showed no command following, and the gag reflex was absent.

Palliative Care determined the prognosis was reserved and, after ethics committee review and appointment of a surrogate, care was transitioned to comfort measures only. Eight days after ICU entry, he remained ventilator-dependent and on low-dose vasopressors, with severe encephalopathy. The patient died 13 days after initial admission.

## Discussion

This case illustrates a rare mechanism for the development of ACS in the setting of UGIB, where the rise in IAP was secondary not to intra-abdominal bleeding, but to a combination of diffuse capillary leak following massive transfusion and bowel distension due to intraluminal hemorrhage. 

While most reports describe ACS as a result of hemoperitoneum or retroperitoneal hematoma, this case highlights a distinct pathophysiologic process. Reports of ACS following UGIB are scarce in the literature. Tchantchaleishvili et al. described a patient with a duodenal diverticulum who developed ACS exclusively secondary to intraluminal hemorrhage [8]. In contrast, our case demonstrates a unique mechanism in which IAH was caused by the combined effects of capillary leak within the peritoneal cavity and intraluminal hemorrhage. This emphasizes the importance of maintaining a high index of suspicion for transfusion-related ACS when increasing resuscitation requirements are accompanied by unexplained ventilatory deterioration.

Even with the availability of endoscopic interventions and pharmacologic therapy, UGIB remains a severe emergency with mortality rates still ranging from 5-14%, especially higher in variceal bleeding [9-11]. MTPs, emphasizing rapid, balanced replacement of red cells, plasma, and platelets, are crucial to stabilize hemodynamics and improve survival in patients with active, exsanguinating hemorrhage [12,13]. However, they carry significant risks, including coagulopathy, electrolyte and acid-base disturbances, hypothermia, transfusion-related lung injury, infection, and multi-organ dysfunction [11,14], underscoring the need for judicious use once bleeding control is achieved.

ACS is a severe complication that develops from IAH, may affect up to 50% of critically ill patients, and is closely related to polytransfusion [15]. According to the World Society of the Abdominal Compartment Syndrome (WSACS), IAH is graded from I to IV based on IAP (grade I: IAP 12-15 mmHg; grade II: IAP 16-20 mmHg; grade III: IAP 21-25 mmHg; and grade IV: IAP >25 mmHg), while ACS is defined as sustained IAP >20 mmHg with new organ dysfunction [5]. Reported incidence varies by population: in trauma patients, ACS develops in up to 14%, while rates may exceed 30% in severe acute pancreatitis and burn patients [16]. 

Etiologically, ACS is subclassified as primary, when due to intra-abdominal causes such as trauma, hemorrhage, or pancreatitis. It can also be classified as secondary, when triggered by extra-abdominal insults like sepsis, burns, or massive resuscitation, and recurrent or tertiary, when the syndrome redevelops after previous treatment and apparent resolution [5]. Diagnosis is primarily clinical, but the sensitivity of physical examination is low, making serial bladder pressure monitoring the recommended method.

Sustained elevations in IAP compromise venous return and cardiac output, reduce renal perfusion, and impair diaphragmatic excursion. Clinically, ACS often presents with nonspecific findings and may include progressive abdominal distension, hemodynamic instability, oliguria, and increased ventilatory pressures with hypoxemia or hypercapnia [15,16], as was seen in this patient. Because these findings can mimic or overlap with the progression of the primary disease process, early recognition is challenging, underscoring the importance of vigilant monitoring.

ACS is typically managed within multidisciplinary surgical and critical care teams, most commonly in trauma and intensive care unit settings. Within this collaborative framework, anesthesiologists play a critical role in perioperative and intraprocedural management, as they are often the first to identify cardiovascular or ventilatory deterioration that may signal the development of ACS. In this case, which occurred in an IR suite, the anesthesiologist identified a pattern of progressive ventilatory failure and initiated the evaluation that led to early communication with surgical teams, timely measurement of IAP, and surgical decompression, which are essential to prevent progression to overt ACS [16].

The management of ACS requires a progressive and multidisciplinary strategy, with the goal of reducing IAP and preventing irreversible organ dysfunction. Initial measures should prioritize careful fluid balance, gastric and colonic decompression, and the improvement of abdominal wall compliance through sedation or neuromuscular blockade [15,16]. As excessive fluid resuscitation has been identified as one of the most significant risk factors for secondary ACS, emphasizing the need for individualized, goal-directed resuscitation and timely de-resuscitation protocols, plays a significant role in management [17,18]. 

When conservative approaches are insufficient, escalation to invasive strategies such as percutaneous drainage or, ultimately, decompressive laparotomy becomes necessary. Although laparotomy is the definitive treatment capable of rapidly restoring perfusion, its timing remains critical. In some conditions, such as acute pancreatitis, thresholds of 25-30 mmHg may justify intervention, while in a ruptured abdominal aortic aneurysm, earlier decompression is recommended [5,19]. Therefore, successful management of ACS relies not only on technical intervention but also on early recognition of high-risk patients, judicious fluid management, and coordinated multidisciplinary decision-making. In the present case, however, at the time ACS was suspected, IAP was already critically elevated (>85 mmHg), accompanied by refractory ventilatory failure, precluding the use of conservative measures and necessitating immediate surgical decompression.

Postoperative management of ACS centers on open abdomen strategies, with temporary closure, planned re-explorations, and early primary fascial closure ideally within the first week to reduce complications. 

Prognosis is highly dependent on timing, as delayed decompression is associated with increased mortality, while prolonged open abdomen increases the risks of infection, fistula, and frozen abdomen [18]. Outcomes are also shaped by etiology, as survival varies between pancreatitis, ruptured aneurysm, and severe burns, with thresholds and timing of intervention differing accordingly [19]. Overall, early recognition, timely decompression, and disciplined ICU management remain essential for improving outcomes, although prognosis remains guarded.

## Conclusions

This case highlights an uncommon presentation of ACS secondary to diffuse capillary leak following massive transfusion and intraluminal hemorrhage in an elderly patient with UGIB. It underscores that, beyond hemorrhagic or traumatic causes, excessive fluid and blood product resuscitation can also precipitate critical IAH and ventilatory failure. Early recognition of unexplained rises in airway pressure, prompt measurement of IAP, and coordinated multidisciplinary intervention are essential to prevent irreversible organ dysfunction. Awareness of transfusion-related ACS should therefore be maintained during massive resuscitation to ensure timely diagnosis and improve patient outcomes.
